# First report of KPC variants conferring ceftazidime-avibactam resistance in Colombia: introducing KPC-197

**DOI:** 10.1128/spectrum.04105-23

**Published:** 2024-05-03

**Authors:** Elsa De la Cadena, María Fernanda Mojica, Laura J. Rojas, Betsy E. Castro, Juan Carlos García-Betancur, Steven H. Marshall, Natalia Restrepo, Nancy Patricia Castro-Caro, Magalis Fonseca-Carrillo, Christian Pallares, Robert A. Bonomo, María Virginia Villegas

**Affiliations:** 1 Grupo de Investigación en Resistencia Antimicrobiana y Epidemiología Hospitalaria, Universidad El Bosque, Bogotá, Colombia; 2 Department of Molecular Biology and Microbiology, School of Medicine, Case Western Reserve University, Cleveland, Ohio, USA; 3 CWRU-Cleveland VAMC Center for Antimicrobial Resistance and Epidemiology (Case VA CARES), Cleveland, Ohio, USA; 4 Research Service, VA Northeast Ohio Healthcare System, Cleveland, Ohio, USA; 5 Fundación Hospital San Vicente de Paul, Rionegro, Colombia; 6 Clínica Farallones Christus Sinergia, Cali, Colombia; 7 Department of Medicine, Case Western Reserve University School of Medicine, Cleveland, Ohio, USA; 8 Louis Stokes Cleveland Department of Veterans Affairs Medical Center, Cleveland, Ohio, USA; 9 Department of Biochemistry, Case Western Reserve University School of Medicine, Cleveland, Ohio, USA; 10 Department of Pharmacology, Case Western Reserve University School of Medicine, Cleveland, Ohio, USA; 11 Department of Proteomics, Case Western Reserve University School of Medicine, Cleveland, Ohio, USA; 12 Department of Bioinformatics, Case Western Reserve University School of Medicine, Cleveland, Ohio, USA; Johns Hopkins University, Baltimore, Maryland, USA

**Keywords:** ceftazidime/avibactam, KPC variants, *Klebsiella pneumoniae*, whole genome sequencing

## Abstract

Resistance to ceftazidime-avibactam (CZA) due to *Klebsiella pneumoniae* carbapenemase (KPC) variants is increasing worldwide. We characterized two CZA-resistant clinical *Klebsiella pneumoniae* strains by antimicrobial susceptibility test, conjugation assays, and WGS. Isolates belonged to ST258 and ST45, and produced a KPC-31 and a novel variant KPC-197, respectively. The novel KPC variant presents a deletion of two amino acids on the Ω-loop (del_168–169_EL) and an insertion of two amino acids in position 274 (Ins_274_DS). Continued surveillance of KPC variants conferring CZA resistance in Colombia is warranted.

**IMPORTANCE:**

Latin America and the Caribbean is an endemic region for carbapenemases. Increasingly high rates of *Klebsiella pneumoniae* carbapenemase (KPC) have established ceftazidime-avibactam (CZA) as an essential antimicrobial for the treatment of infections due to MDR Gram-negative pathogens. Although other countries in the region have reported the emergence of CZA-resistant KPC variants, this is the first description of such enzymes in Colombia. This finding warrants active surveillance, as dissemination of these variants could have devastating public health consequences.

## OBSERVATION

*Klebsiella pneumoniae* carbapenemase (KPC) is the most globally widespread carbapenemase (1). KPC-producing Gram-negatives are often resistant to most β-lactams but remain susceptible to new β-lactam/β-lactamase inhibitors, such as ceftazidime-avibactam (CZA) (2). The most common mechanism of resistance to CZA is the production of metallo-β-lactamases (MBL) since they are not inhibited by avibactam (3). However, an increasingly worrisome number of new KPC variants conferring resistance to CZA are being found in *Enterobacterales* in clinical settings (4, 5). Resistance to CZA in KPC-producing *K. pneumoniae* is often related to *bla*_KPC-2_ or *bla*_KPC-3_ variants, as well as *bla*_KPC_ overexpression and changes in membrane permeability due to loss of porins or increased efflux pump expression (6, 7). KPC variants conferring CZA resistance have substitutions, insertions, and/or deletions in various *hot spots*, including the Ω -loop region, the loop 237–243, and the loop 266–275 helix (8).

Two *K. pneumoniae* clinical isolates that displayed CZA resistance were collected in two Colombian cities, Cali (Kpn-991, abdominal secretion) and Rionegro (Kpn-1001, suprahepatic collection) during 2023 as part of an on-going CZA resistance surveillance study. Per standard protocol, the antimicrobial susceptibility was confirmed by broth microdilution (BMD) using customized panels (Sensititre panels; TREK Diagnostic Systems, Thermo Fisher, UK) and interpreted according to the Clinical and Laboratory Standards Institute (CLSI) 2023 (9). CZA susceptibility was further evaluated by E-test (bioMerieux, Marcy l'Etoile, France). As shown in Table 1, isolates Kpn-991 and Kpn-1001 were resistant to CZA and susceptible to imipenem, meropenem, meropenem/vaborbactam, and imipenem/relebactam. Isolate Kpn-1001 was also susceptible to ertapenem, while Kpn-991 was resistant to this agent. Following standard procedure, the isolates were then subjected to carbapenemase screening by in-house CLSI Carba NP test (9), which yielded a negative report for carbapenemase activity. Surprisingly, the presence of *bla*_KPC_ was revealed by the in-house real-time quantitative polymerase chain reaction (qPCR) method (10), and production of KPC was further confirmed using a NG-Test CARBA 5 (NG Biotech, Guipry, France).

**TABLE 1:** Antimicrobial susceptibility for CZA-resistant *K. pneumoniae*, TC, and *E. coli* J53^*a*^

Antimicrobial agent	MIC (mg/L)			
	Kpn-991	Kpn-1001	*E. coli* J53 TC1001	*E. coli* J53
Ceftazidime	>128	>128	64	≤1
Cefepime	>16	>16	8	≤1
Piperacillin/tazobactam	>64/4	32/4	16-abr	≤4/4
Aztreonam	>16	>16	>16	≤1
Ertapenem	64	≤0.5	≤0.5	≤0.5
Imipenem	≤0.5	≤0.5	≤0.5	≤1
Meropenem	≤0.5	1	≤1	≤1
Ceftazidime/avibactam	32/4	32/4	32/4	≤2/4
Meropenem/vaborbactam	≤1/8	≤1/8	≤1/8	≤1/8
Imipenem/relebactam	≤0.5/4	≤0.5/4	≤0.5/4	≤0.5/4
Amikacin	16	≤8	≤8	≤8
Gentamicin	>8	≤4	≤4	≤4
Trimethoprim/sulfamethoxazole	>4/76	>4/76		≤2/38
Colistin	≤0.5	≤0.5	≤0.5	≤0.5
Tigecycline	≤1	≤1	≤1	≤1
Fosfomycin	≤32	≤32	≤32	≤32

^*a*^ TC: transconjugant.

**Fig 1:**
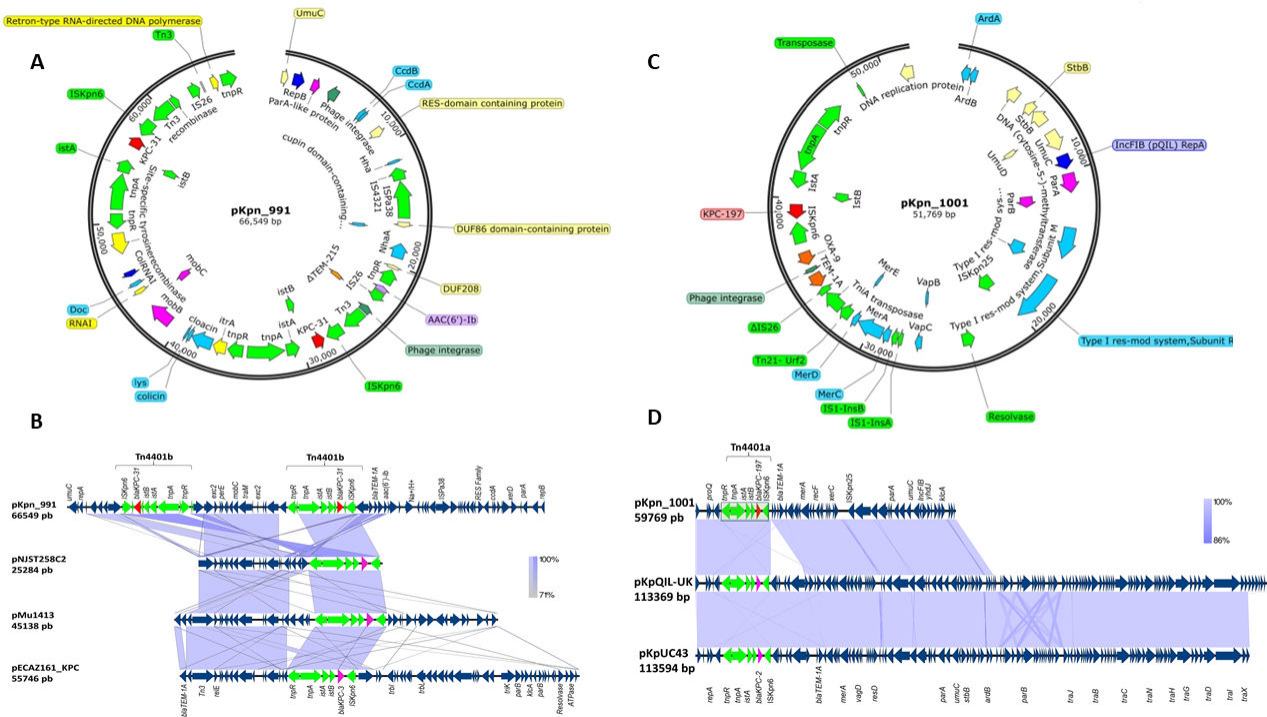
Gene maps of *bla*_KPC-31_-containing repB/ColRNAI plasmid (**A**), comparative genomic analysis of related plasmids identified in *K. pneumoniae* strain 991 harboring two copies of the *bla*_KPC-31_ gene in the Tn*4401b* (green color). The sequences compared are: pNJST258C2 (GenBank: CP006919.1), pMu1413 (GenBank: CP096817.1), and pECAZ161_KPC (GenBank: CP019010.1) that harbor *bla*_KPC-3_. The shaded area between sequences delimits regions of alignment with percent identity ≥71%. Dark blue arrows indicate open reading frames, *bla*_KPC-3_ gene is in purple, and *bla*_KPC-31_ is in red (**B**). New *bla*_KPC-197_-variant- containing IncFIB(pQil) plasmid (**C**), comparative genomic analysis of related plasmids identified in *K. pneumoniae* strain 1001 harboring *bla*_KPC-197_ gene in the Tn*4401a* (green color). The sequences compared are: plasmids pKpQIL-UK (GenBank: KY798507.1) and pKpUC43 (GenBank: CP115931.1) that harbor *bla*_KPC-2._ The shaded area between sequences delimits regions of alignment with percent identity ≥86%. Dark blue arrows indicate open reading frames, *bla*_KPC-3_ gene is in purple, and *bla*_KPC-197_ is in red (**D**).

To investigate the CZA resistance mechanism, isolates were subjected to WGS analysis via both short- and long-read sequencing using MiSeq (Illumina, San Diego, CA) and Oxford Nanopore MinION (Oxford Nanopore Technologies, Oxford, UK). For the Illumina generated reads, *de novo* assemblies were generated using Spades v3.13 and annotation of predicted genes was performed using BV-BRC platform (https://www.bv-brc.org/). The long reads were *de novo* assembled with Flye 2.9, polished with Medaka 1.6, and annotated using BV-BRC (Bacterial and Viral Bioinformatics Resource Center) (11). WGS analysis showed that Kpn-991 belongs to ST258 and produces KPC-31, a KPC-3 variant possessing the D179Y substitution (4). *bla*_KPC-31_ was located within a transposon Tn*4401*b. Detailed analysis demonstrated that Kpn-991 harbors two copies of *bla*_KPC-31_ within a repB/ColRNAI plasmid (66.5 Kb) (Table 2). In turn, Kpn-1001 belongs to ST45 and harbors a new KPC-3 variant recently designated as KPC-197 located within Tn*4401*a, on an IncFIB(pQil) plasmid (51.7 Kb) (Fig. 1).

**TABLE 2:** Genetic characterization of strains *K. pneumoniae* 991 and 1001

	Kpn-991	Kpn-1001
MLST	258	45
KPC variant	31	197
Chromosome size (PB)	5.737.061	5.498.646
Resistance genes	*bla*_SHV-11_, *bla*_CTM-M-15_, *bla*_OXA-1_, aph (6)-ld, aac (3)-IIa, aph(3´´)-Ib, qnrB1, catB3, tet(A), fosA, Sul2, dfrA17	*bla*_TEM-1_, *bla*_SHV-1_, *bla*_CTX-M-15_, *bla*_OXA-1_, *bla*_OXA-9_, aph (6)-Id, aph(3´´)-Ib, aac(6´)-Ib-cr, *qnrB*q, catB3, fosA, dfrA14
Virulance genes	Colibactim: *Clb*A-I/L-R, Yersiniabactin: *fyuA/ irp/ ybt*, Type 3 fimbriae: *mrk* ABCDFHIJ, *iutA*	Yersiniabactin: fyuA/ irp/ybt, Type 3 fimbriae: mrk ABCDFHIJ, *iutA*
Inc type	IncFII (Yp), IncFIB (K), IncFII (K), ColRNAI, repB	IncFIB(K), IncFIB(pQil), IncFII(K),
*ompK35* profile	Wild type	Frameshift mutation (amino acid 96), premature stop codon (amino acid 102)
*ompK36* profile	GD insertion at amino acids 134–135	Wild type

Plasmid sequence BLAST against NCBI GenBank (http://blast.ncbi.nlm.nih.gov/Blast.cgi) showed that pKpn_1001_KPC-197 is highly similar to two plasmids harboring *bla*_KPC-2_, pKpQIL-UK (GenBank: KY798507.1), and pKpUC43 (GenBank: CP115931.1), from two *K. pneumoniae* isolates collected in the United Kingdom and in the United States, respectively. Likewise, pKpn_991_KPC-31 is very similar to three plasmids that harbor *bla*_KPC-3_, pNJST258C2 (GenBank: CP006919.1), pMu1413 (GenBank: CP096817.1), and pECAZ161_KPC (GenBank: CP019010.1) (Fig. 1). These plasmids were recovered from two *K. pneumoniae* (pNJST258C2 and pMu1413) and one *Escherichia coli* isolate from the United States. Further conjugation assays using *E. coli* J53 as the recipient strain demonstrated that the plasmid containing the *bla*_KPC_ gene was transferable from Kpn-1001 but not Kpn-991, as confirmed by qPCR performed on transconjugant TC1001. Consistent with the successful transfer and expression of *bla*_KPC_, TC1001 displayed an MIC of 32 mg/L for CZA, remaining susceptible to imipenem and meropenem (Table 1).

**Fig 2:**
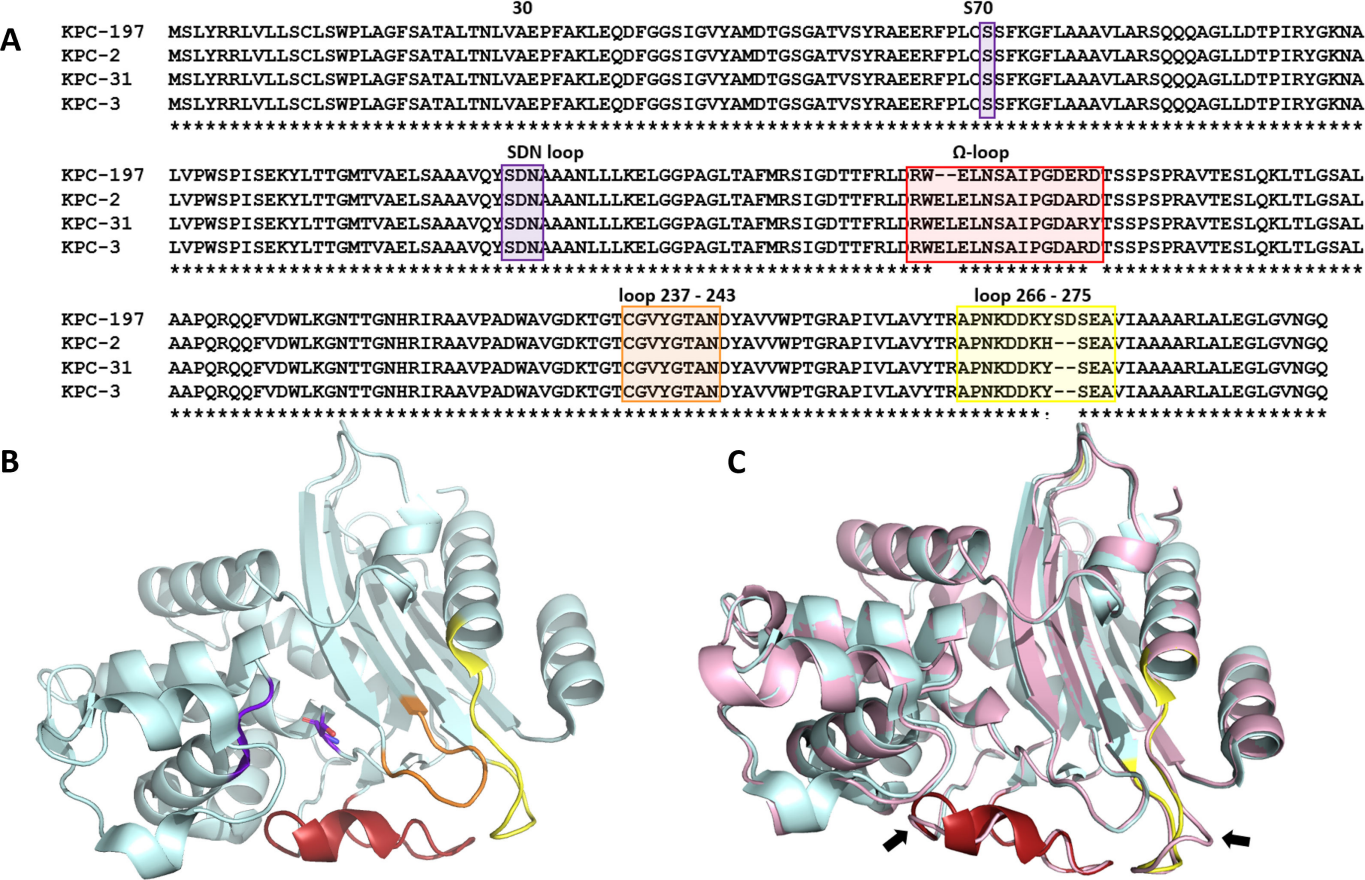
(**A**). Multiple sequence alignment of the amino acid sequence of representative KPC variants and the KPC-197 found in isolate Kpn_1001. Regions of interest for development of CZA resistance, the Ω-loop, loop 237–243, and the loop 266–275 helix are highlighted in red, orange, and yellow, respectively. The active site Ser70 as well as the SDN loop are highlighted in purple. (**B**) Crystal structure of KPC-2 (PDB 2OV5) with the CZA resistance hot spots highlighted as in (A). (**C**) Overlay of KPC-2 (light cyan) with a model of the new KPC-197 found in Kpn_1001 generated by SWISS-MODEL (https://swissmodel.expasy.org/) shown in pink. The arrows point to the predicted structural changes in the Ω- and 266–275 loops of the novel KPC-197 variant.

The novel KPC-197 harbors an A177E substitution, a deletion of two amino acids on the Ω-loop (del_168–169_EL), and an insertion of two amino acids in position 274 (Ins_274_DS) (Fig. 2A and C). The deletion of the EL residues at position 167 was previously reported in KPC-66 (amino acid sequence identity, 98.98%) and KPC-92 (98.63%) among others, and the Ins_274_DS has been previously reported in KPC-82 (98.64%) (8, 12). Comprehensive biochemical characterization of this novel KPC-197 is currently being undertaken. The sequencing data have been deposited in GenBank database under BioProject number PRJNA1001578 and accession number OR633287 for KPC-179 variant.

Numerous variants of KPC conferring resistance to CZA have been described among clinical isolates worldwide (8). The first CZA-resistant isolate was reported in 2015 in the United States. This *K. pneumoniae* harbored porin mutations with increased *bla*_KPC-3_ expression (13). The first report in South America occurred in 2020 with the identification of three CZA-resistant *K. pneumoniae* isolates ST11 harboring KPC-8 in Argentina (14). Recently, KPC-113 and KPC-114 were identified from *K. pneumoniae* isolates belonging to ST11 and ST16 in Brazil (15). KPC-31 has been reported in Italy, the United States, and Argentina (14–16). As also shown here, this KPC variant confers resistance to CZA at the expense of the carbapenemase activity (7, 16). Interestingly, Faccone et al. identified a KPC-31 in a CZA-resistant *Pseudomonas aeruginosa* ST235, which had been exposed to CZA (17). Indeed, previous studies have shown that prolonged exposure to CZA selects CZA-resistant isolates producing KPC variants (18)

Colombia is a country endemic for KPC, where the co-circulation of KPC-2 and KPC-3 in *Enterobacterales* has been well-documented (19–21). A variety of *K. pneumoniae* sequence types has been associated with the dissemination of *bla*_KPC-2_; simultaneously, isolates belonging to the “high risk clone” CG258 have been found to be the main drivers of *bla*_KPC-3_ spread (19). Of note, ST45, which one of the isolates described in this study belonged to, has been reported in other studies carrying *bla*_KPC-3_ and *bla*_NDM-1_ in Colombia (21, 22). As far as mobile genetic elements, the most common plasmid replicons in *bla*_KPC_-harboring isolates are IncFIB(K), IncFII(K), ColRNAI, IncR, and IncFII. Although Tn*4401* transposon remains the most frequent element within most isolates, a study by Saavedra et al. reported a variety of other mobile genetic elements carrying *bla*_KPC_ (21).

In this context, CZA was a long-awaited addition to the armamentarium against carbapenem-resistant Gram-negatives. CZA therapy was approved by the Colombian health regulatory authorities in 2019. A previous surveillance study conducted on strains collected before the introduction of CZA in Colombia found a 5.7% of resistance to this combination, exclusively due to the production of MBLs in *Enterobacterales* (23, 24). Therefore, this is the first report of CZA resistance in *Enterobacterales* due to the production of CZA-resistant KPC variants in Colombia.

The two isolates producing KPC variants recovered in our study exhibited resistance to CZA; conversely, they also exhibited susceptibility to imipenem and meropenem, and to the newer β-lactam/β-lactam inhibitor combinations meropenem/vaborbactam and imipenem/relebactam. A trade-off between CZA resistance and decreased carbapenemase activity is often observed among these KPC variants (8). On one hand, this feature makes detection of these KPC variants challenging. As the Carba NP test detects the *in vitro* hydrolysis of imipenem by a bacterial lysate using the pH indicator phenol red (10), KPC variants with impaired imipenem hydrolysis will not be detected. Thus, molecular tests as PCR or immunoassays such as NG-Test CARBA 5 are needed to track their dissemination. On the other hand, this feature might offer an opportunity for patients infected by CZA-resistant KPC-producing *Enterobacterales* to be treated with imipenem/relebactam and meropenem/vaborbactam (25). Nevertheless, a marked overproduction of KPC associated with impairment of major porins may led to development of cross-resistance to ceftazidime/avibactam, meropenem/vaborbactam, and imipenem/relebactam in KPC-producing *Klebsiella pneumoniae* isolates (26, 27).

In conclusion, the detection of *K. pneumoniae* isolates resistant to CZA due to the production of KPC-3 variants in two different cities in Colombia is worrisome. Moreover, it warrants thorough surveillance studies with accurate identification of KPC variants conferring CZA resistance. If the findings described here are only the “tip of the iceberg” and the CZA resistance in the country is more widespread than previously known, the use of this antibiotic as an alternative therapeutic could be severely compromised.
